# Supplementary material for: Comprehensive Analysis of the Prognostic Value and Molecular Function of CRNDE in Glioma at Bulk and Single-Cell Levels
Source: Cells. 2022 Nov 18;11(22):3669. doi: 10.3390/cells11223669 (PMC9688829; doi:10.3390/cells11223669)

Supplementary Materials

Table S1. The sequences of ASO and primers.

Name	Sequence
ASO-CRNDE-1	5'-GAAATTCATCCCAAGGCTGG-3'
ASO-CRNDE-2	5'-GGGTTCCTCCAAATGTTGGC-3'
CRNDE forward	5'-ATTTGGATGCTGTCAGCTAAGTTC-3'
CRNDE reverse	5'-GACCAGCCTTGGGATGAATTTC-3'
GAPDH forward	5'-ACCCACTCCTCCACCTTTGAC-3'
GAPDH reverse	5'-TGTTGCTGTAGCCAAATTCGTT-3'
ACTB forward	5'-CATGTACGTTGCTATCCAGGC-3'
ACTB reverse	5'-CTCCTTAATGTCACGCACGAT-3'
α-tubulin forward	5'-GAGTGCATCTCCATCCACGTT-3'
α-tubulin reverse	5'-TAGAGCTCCCAGCAGGCATT-3'

Figure S1. The ROC curve based on the expression of CRNDE to predict the 5-year OS of LGG patients in TCGA. The optimum cutoff value of CRNDE was 0.2757 with a specificity of 0.7341 and a sensitivity of 0.7179.

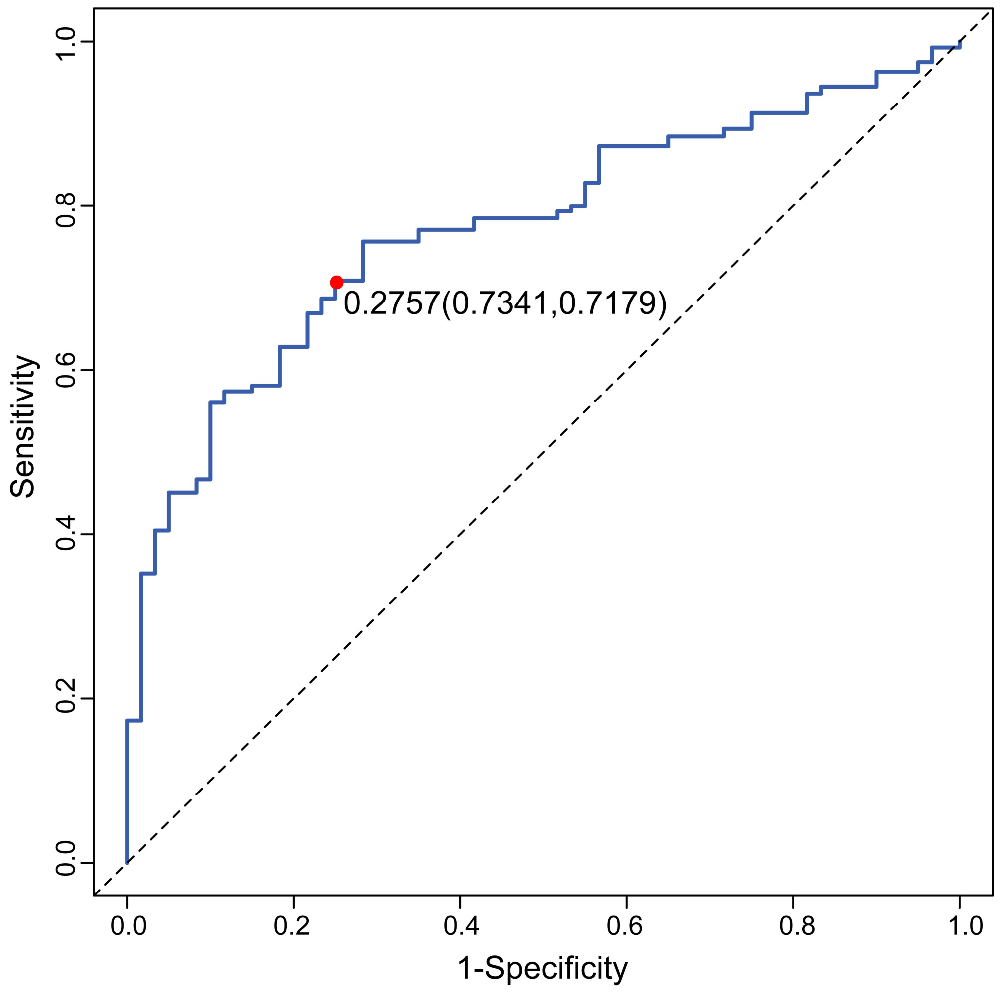

Figure S2. Association of clinical and molecular factors with OS based on univariate and multivariate Cox analyses of all glioma patients in TCGA, CGGA, and the whole dataset.

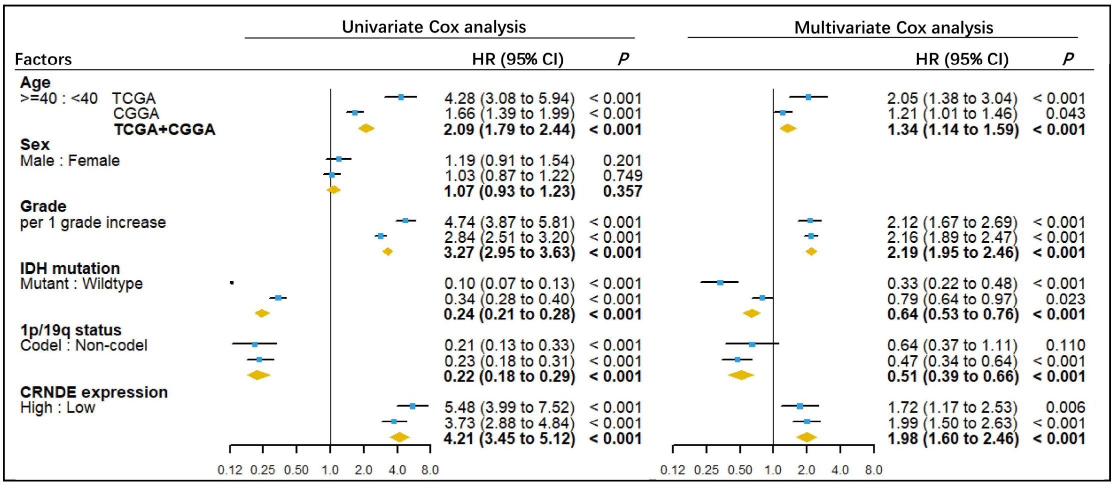

Figure S3. The expression features of marker genes of the four major clusters. tSNE plot of the 5311 cells with each cell color-coded by the expression level of marker genes of glioma cell **(A)**, myeloid cell **(B)**, oligodendrocyte **(C)**, and T cell **(D)**.

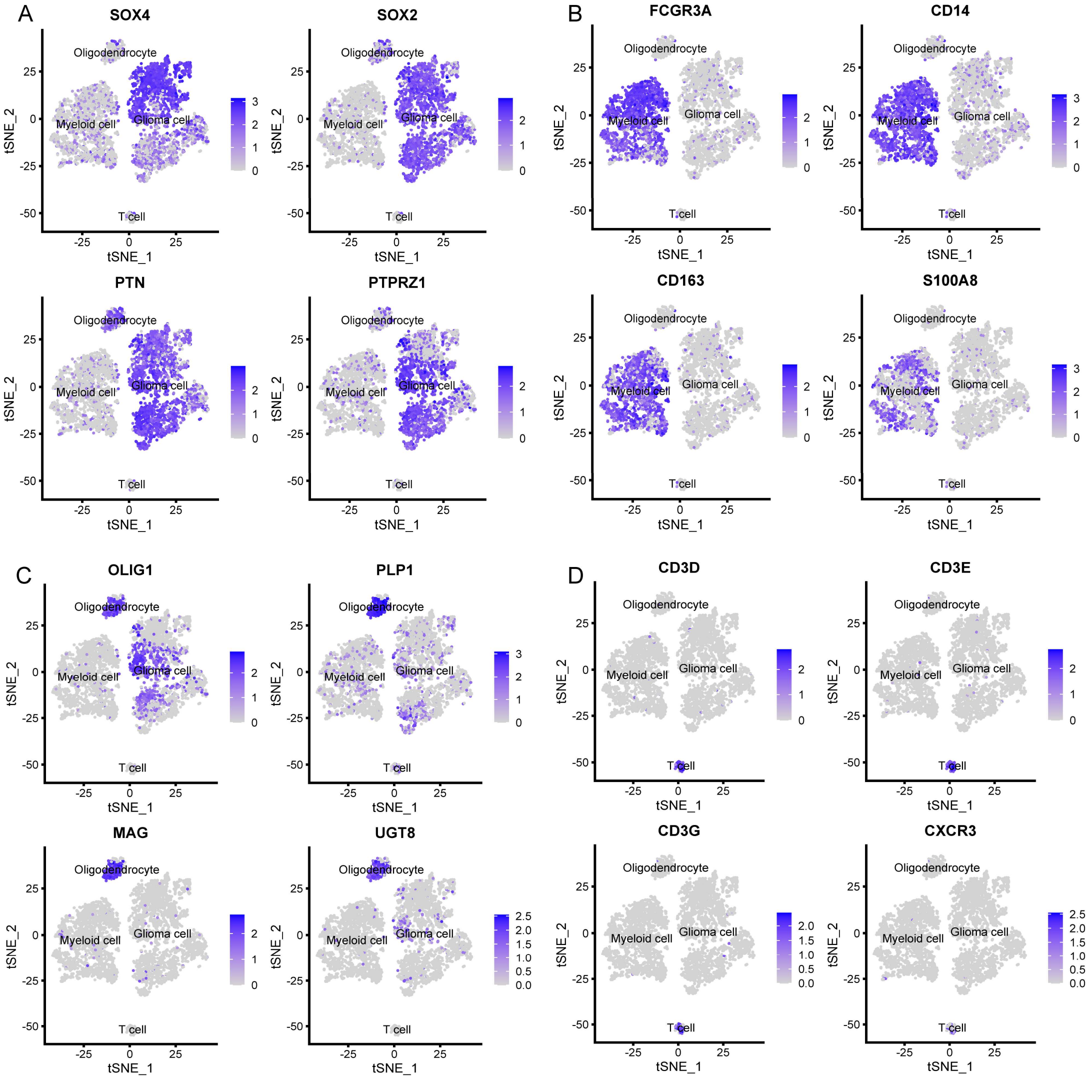

Figure S4. Knockdown efficiency of CRNDE in U118 and SW1783 verified by RT-qPCR. ACTB **(A)**, α-tubulin **(B)**, and GAPDH **(C)** were used as endogenous controls. (**P < 0.01)

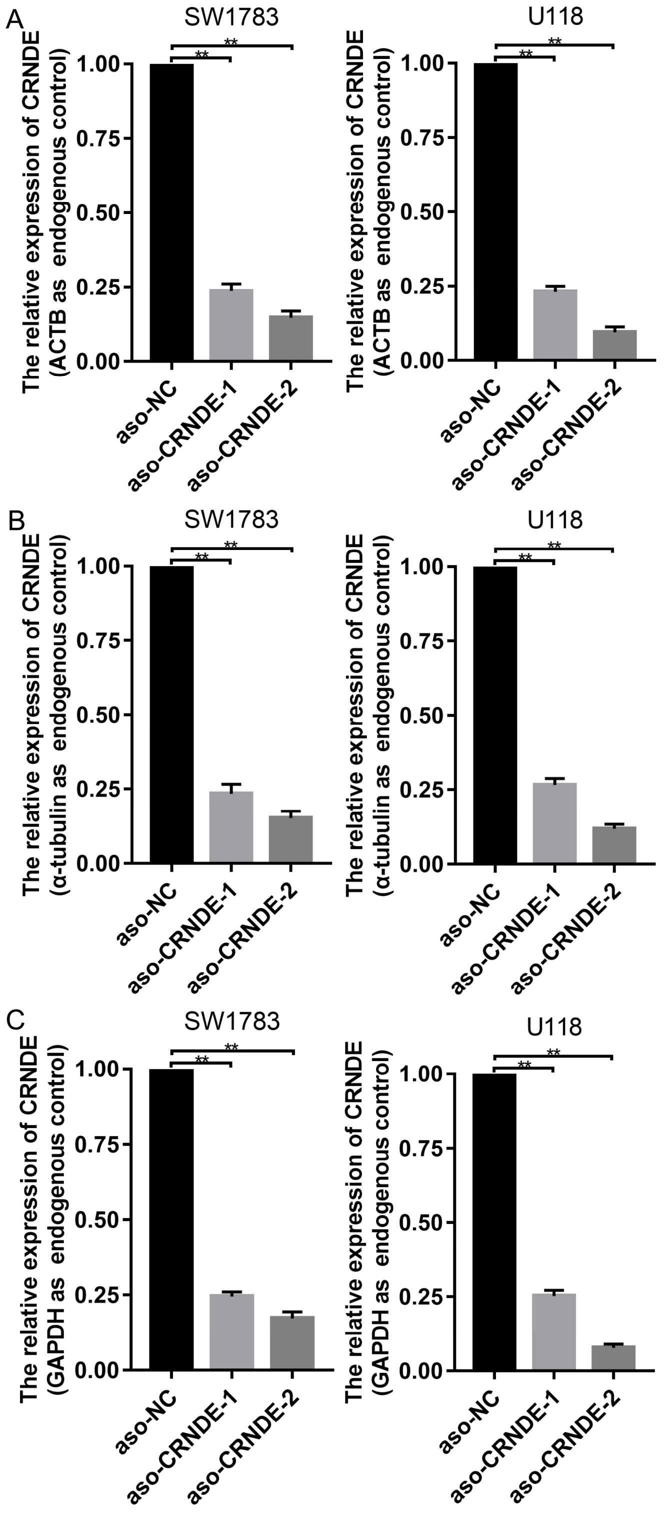

Supplement: Supplementary file 1 [file cells-11-03669-s001.zip › cells-2008180-supplementary.pdf]
